# Impact of the COVID-19 pandemic on breast cancer diagnosis and treatment trends in Japan

**DOI:** 10.1007/s12282-025-01718-2

**Published:** 2025-05-12

**Authors:** Minoru Miyashita, Hiraku Kumamaru, Naoki Hayashi, Fuyo Kimura, Hiroyuki Yamamoto, Naoki Niikura, Yasuaki Sagara, Hiromitsu Jinno, Masakazu Toi, Shigehira Saji

**Affiliations:** 1 Department of Breast and Endocrine Surgical Oncology, Tohoku University School of Medicine, 1-1 Seiryo-Machi, Aoba-Ku, Sendai, 980-8574 Japan; 2 Department of Healthcare Quality Assessment, University of Tokyo, 7-3-1 Hongo, , Bunkyo-Ku, Tokyo 113-8655 Japan; 3 Division of Breast Surgical Oncology, Department of Surgery, Showa University School of Medicine, Tokyo, Japan; 4 Department of Breast, Tokyo Medical University Hospital, 6-7-1 Shinjuku, Shinjuku-ku, Tokyo 160-0023 Japan; 5 Department of Breast Oncology, Tokai University School of Medicine, 143 Shimokasuya, Isehara, Kanagawa 259-1193 Japan; 6 Department of Breast Surgical Oncology, Social Medical Corporation Hakuaikai, Sagara Hospital, 3-28 Matsubara, Kagoshima, 892-833 Japan; 7 Department of Surgery, Teikyo University School of Medicine, 2-11-1 Kaga, Itabashi-ku, Tokyo, 173-8606 Japan; 8 Tokyo Metropolitan Komagome Hospital, 3 Chome-18 Honkomagome, Bunkyo City, Tokyo Japan; 9 Department of Medical Oncology, Fukushima Medical University, Fukushima, Japan

**Keywords:** COVID-19, Pandemic, Breast cancer, Japanese Breast Cancer Society, Registry, National clinical database

## Abstract

**Background:**

There is no comprehensive report regarding which patient groups were disrupted by the COVID-19 pandemic in Japan having universal health insurance system. To provide the guidance regarding how to act in future pandemics, we investigated the changes in breast cancer (BC) diagnosis and treatment during the COVID-19 pandemic.

**Methods:**

The trends of monthly data were calculated in relation to the variables of a total of 291,018 primary BCs registered on the Japanese National Clinical Database between January 2018 and April 2021.

**Results:**

An analysis of the nationwide data during the pandemic showed 9% decrease of newly identified BC compared with before the pandemic. The impact was more relevant in the 40–50, 51–60 and 61–70-years age groups (13%, 8% and 9% decrease, respectively). The most substantial reduction was noted in patients identified through screenings without symptoms with a 17% decrease. These effects were also apparent in cT1, cN0, cStage 0, and cStage I (11%, 9%, 8% and 11% decrease, respectively). In breast surgery procedures, there was a notable decrease in breast-conserving surgery (13%) as well as post-operative radiation therapy (11%). During this period, strategies using neoadjuvant endocrine therapy or chemotherapy were implemented to avoid treatment delays for especially Stage I patients (1.5 folds increase).

**Conclusions:**

We have identified the patient groups that are more vulnerable to the effects of the pandemic. The changes during the pandemic might provide the guidance regarding how to act in future emergencies to minimize disadvantages for BC patients.

**Supplementary Information:**

The online version contains supplementary material available at 10.1007/s12282-025-01718-2.

## Introduction

The first confirmed case of COVID-19 in Japan was identified in January 2020, and similar to the situation in many other countries globally, the infection progressively spread. By April 2020, a state of emergency was declared. There was a call to avoid nonessential outings, and some healthcare services were either partially halted or reassessed. 

The impact of the suspension of services on cancer screenings was substantial, and the screening rates also significantly decreased in Japan, similar to that in other countries [1–6]. In 2020, the number of screenings for five types of cancer—stomach, colon, breast, cervical, and lung—decreased by 27.4% compared with those in 2019. Furthermore, there was a 27.2% decrease in the breast cancer (BC) screening examinations. The decline in BC screening rates, as observed, could potentially affect early detection and treatment, which are primary strategies for reducing cancer mortality rates. Moreover, in Japan, half of the BC cases are detected through self-examination, and there was also concern about decreased visits to healthcare facilities by individuals with symptoms [7]. The decrease in screening participation rates and the reluctance of symptomatic individuals to visit medical facilities suggest potential delays in BC treatment. However, there are no comprehensive reports on the extent of the impact of these factors on a national scale in Japan. 

There have been more advancements in drug therapy for BC compared with other types of cancer. It may be feasible to alleviate delays in diagnosis and surgical waiting times, to some extent, by applying neoadjuvant endocrine therapy or chemotherapy. Furthermore, while BC is typically perceived to progress at a slow pace, the rate of progression can differ based on its subtypes [8, 9]. For instance, the progression of luminal-type BC, which is hormone receptor-positive, estrogen receptor (ER)-positive and/or progesterone receptor (PgR)-positive, and human epidermal growth factor receptor 2 (HER2)-negative is usually slow. However, in triple-negative BC, which is hormone receptor-negative and HER2-negative, the progression can be swift, and diagnostic delays could potentially affect the treatment outcomes [10]. Despite the unique circumstances and biological features surrounding BC, the effects of the COVID-19 pandemic on this disease are still entirely unknown.

Japan has established a healthcare system with relatively easy access to medical care and hospitalization based on the national health insurance system and a higher number of hospital beds per capita than other countries. In this context, a comprehensive review of the impact of the COVID-19 pandemic on the diagnosis and treatment of BCs that occurred during this period is essential. In the event of a future emergency, such as the declaration of a state of emergency, this study aims to provide a reference for taking the appropriate response measures. Specifically, we here examined how BC screenings and treatments should be conducted. Using the National Clinical Database-Breast Cancer Registry (NCD-BCR) data [11], we investigated the changes in BC diagnosis and treatment before and during the COVID-19 pandemic.

## Materials and methods

### Data source

This study was conducted using the NCD-BCR data that retrospectively collects primary data on BC from approximately 1,400 medical facilities across Japan [11]. In the cases where surgery is conducted, nearly 100% of BC cases in Japan are registered in this database. In the NCD-BCR, approximately 100,000 cases of breast cancer are recorded each year. All BC cases registered in the NCD-BCR are entered voluntarily by the responsible personnel at each institution. Since this database is used for the certification of clinical professionals, the data entered is extremely accurate. This database includes comprehensive patient background information such as location, age, and comorbidities. It also provides extensive clinical and pathological details, including the method of detection, clinical T status, clinical N status, clinical Stage, and subtype. Furthermore, it covers all treatment data, ranging from surgical methods to drug and radiation therapies, including prognosis.

### Study population

This study initially included a total of 291,018 cases of primary BC that were registered on the NCD-BCR, with treatment initiated between January 2018 and April 2021. This period includes the time before and during the COVID-19 pandemic. In this study, we excluded males, individuals who were ≤ 19 years old, and those with simultaneous or metachronous bilateral BC.

We analyzed the monthly trends in the number of BC cases, both on a national scale and within three separate epidemiological region The categorization of epidemiological regions was determined according to the cumulative number of COVID-19 cases in each Japanese prefecture up to December 31, 2020. Regions with a high cumulative number of COVID-19 cases included Tokyo, Okinawa, Osaka, Hokkaido, Kanagawa, Aichi, Saitama, Hyogo, Chiba, Kyoto, Fukuoka, and Nara. Regions with a low cumulative number included Akita, Tottori, Niigata, Tokushima, Shimane, Iwate, Kagawa, Ehime, Yamagata, Aomori, Yamaguchi, Nagasaki, and Fukui. The remaining prefectures were classified as medium areas [12].

### Analytical variables

The trends of monthly data were calculated in relation to age, comorbidities, detection circumstances (Self detection, Screening with symptom, Screening without symptom, Others), clinical T (cT), clinical N (cN), clinical Stage (cStage), BC subtype, surgical methods, neoadjuvant endocrine therapy, (neo) adjuvant chemotherapy, and postoperative radiation therapy. Furthermore, it is important to note that the number of BC cases inherently shows a seasonal pattern, which can be influenced by factors such as the timing of screenings. Typically, fluctuations in the number of cases are observed within a given year. We analyzed the patterns by comparing the annual trends for 2018 and 2019, prior to the COVID-19 pandemic, with the trends for 2020, which encompasses the period during the COVID-19 pandemic. In addition to a monthly analysis, a comparative study was conducted examining the number of cases for each factor during the eight months period from May 2020 to December 2020 with the same period in the previous year.

Because of the overwhelming pressure on the healthcare system caused by the pandemic, there was a temporary need to delay BC surgeries across Japan. To account for the prolonged surgical wait times, it was projected that the treatment would shift to neoadjuvant endocrine therapy or chemotherapy in a specific number of cases. Consequently, the monthly variations in the number of cases receiving neoadjuvant chemotherapy for Stage I–III cases and neoadjuvant endocrine therapy for Stage 0–III cases were analyzed and categorized by Stage. Furthermore, regarding surgical procedures, an increase in total mastectomy was predicted to circumvent the need for breast-conserving surgery, which necessitates regular hospital visits for postoperative radiation therapy. Therefore, we investigated the trends in the number of cases according to the type of breast surgery performed.

## Results

### Changes in the number of breast cancer patients before and during COVID-19

Table 1 illustrates the monthly patient counts, broken down nationwide by high, moderate, and low prevalence areas for the 291,018 cases that were registered from January 2018 to April 2021 [12]. Examination of the changes in nationwide patient numbers compared with that in the previous year showed that after the declaration of a state of emergency in April 2020, the subsequent months showed a yearly decrease of 8% in May, 9% in June, and a peak decrease of 13% in August. This trend continued until February 2021. Examination of the high, medium, and low prevalence areas confirmed that the trends in all areas were the same as the national trend. An analysis of the nationwide data for the 8-month period from May 2020 to December 2020, during the COVID-19 pandemic, showed a decrease of 9% compared with that of the previous year (Table 2).

**Table 1:** Monthly trends of breast cancer cases by regions, nationwide, high, medium, or low epidemic areas

	Jan	YoY	Feb	YoY	Mar	YoY	Apr	YoY	May	YoY	Jun	YoY	Jul	YoY	Aug	YoY	Sep	YoY	Oct	YoY	Nov	YoY	Dec	YoY
2018																								
Nationwide	6756	–	6452	–	6912	–	6747	–	6296	–	7203	–	7314	–	7617	–	6530	–	7728	–	7802	–	7161	–
High epidemic area	4003	–	3867	–	4190	–	3999	–	3756	–	4358	–	4346	–	4558	–	3882	–	4565	–	4618	–	4219	–
Medium epidemic area	1976	–	1884	–	1969	–	1996	–	1828	–	2067	–	2176	–	2223	–	1924	–	2267	–	2255	–	2092	–
Low epidemic area	777	–	697	–	749	–	750	–	709	–	777	–	792	–	832	–	722	–	895	–	925	–	850	–
Unknown	0	–	4	–	4	–	2	–	3	–	1	–	0	–	4	–	2	–	1	–	4	–	0	–
2019																								
Nationwide	7347	1.09	7051	1.09	7099	1.03	7453	1.10	6846	1.09	7418	1.03	7400	1.01	7631	1.00	7556	1.16	8492	1.10	8029	1.03	7947	1.11
High epidemic area	4310	1.08	4191	1.08	4239	1.01	4476	1.12	4202	1.12	4536	1.04	4426	1.02	4651	1.02	4573	1.18	5017	1.10	4844	1.05	4661	1.10
Medium epidemic area	2146	1.09	2091	1.11	2110	1.07	2154	1.08	1959	1.07	2065	1.00	2163	0.99	2189	0.98	2144	1.11	2470	1.09	2344	1.04	2358	1.13
Low epidemic area	888	1.14	768	1.10	749	1.00	822	1.10	684	0.96	817	1.05	808	1.02	791	**0.95**	838	1.16	1004	1.12	841	**0.91**	926	1.09
Unknown	3	–	1	–	1	–	1	–	1	–	0	–	3	–	0	–	1	–	1	–	0	–	2	–
2020																								
Nationwide	7884	1.07	7238	1.03	7719	1.09	7587	1.02	6320	**0.92**	6740	**0.91**	7091	0.96	6629	**0.87**	7017	**0.93**	7604	**0.90**	7110	**0.89**	7530	**0.95**
High epidemic area	4746	1.10	4321	1.03	4687	1.11	4506	1.01	3779	**0.90**	3987	**0.88**	4225	**0.95**	4003	**0.86**	4189	**0.92**	4538	**0.90**	4201	**0.87**	4426	**0.95**
Medium epidemic area	2275	1.06	2123	1.02	2184	1.04	2241	1.04	1829	**0.93**	2014	0.98	2045	**0.95**	1907	**0.87**	1999	**0.93**	2200	**0.89**	2089	**0.89**	2272	0.96
Low epidemic area	863	0.97	792	1.03	848	1.13	840	1.02	712	1.04	738	**0.90**	821	1.02	719	**0.91**	828	0.99	865	**0.86**	820	0.98	832	**0.90**
Unknown	0	–	2	–	0	–	0	–	0	–	1	–	0	–	0	–	1	–	1	–	0	–	0	–

2021	Jan	YoY	Feb	YoY	Mar	YoY	Apr	YoY	Total
Nationwide	7232	**0.92**	6661	**0.92**	8079	1.05	7790	1.03	291,018
High epidemic area	4399	**0.93**	4060	**0.94**	4955	1.06	4709	1.05	174,218
Medium epidemic area	2106	**0.93**	1886	**0.89**	2306	1.06	2265	1.01	84,591
Low epidemic area	726	**0.84**	715	**0.90**	818	0.96	815	0.97	32,163
Unknown	1	–	0	–	0	–	1	–	46

Regions with a high prevalence of COVID-19 included Tokyo, Okinawa, Osaka, Hokkaido, Kanagawa, Aichi, Saitama, Hyogo, Chiba, Kyoto, Fukuoka, and Nara. Regions with low prevalence included Akita, Tottori, Niigata, Tokushima, Shimane, Iwate, Kagawa, Ehime, Yamagata, Aomori, Yamaguchi, Nagasaki, and Fukui. The remaining prefectures were classified as medium prevalence areas. 5% or more decrease is marked in bold

**Table 2:** Monthly trends of breast cancer cases by patients and tumor characteristics

	Jan-20	YoY	Feb-20	YoY	Mar-20	YoY	Apr-20	YoY	May-20	YoY	Jun-20	YoY	Jul-20	YoY	Aug-20	YoY	Sep-20	YoY	Oct-20	YoY	Nov-20	YoY	Dec-20	YoY	May-20 to Dec-20	YoY
Total (n)	7884	1.07	7238	1.03	7719	1.09	7587	1.02	6320	**0.92**	6740	**0.91**	7091	0.96	6629	**0.87**	7017	**0.93**	7604	**0.90**	7110	**0.89**	7530	**0.95**	56,041	**0.91**
Age (y)																										
Median (5%−95%)	62 (40–84)	–	61 (40–83)	–	60 (40–84)	–	60 (39–83)	–	59.5 (40–83)	–	62 (40–84)	–	63 (40–85)	–	62 (40–84)	–	62 (40–85)	–	63 (40–84)	–	62 (40–84)	–	61 (40–84)	–	–	**–**
20–40	460	1.13	366	**0.81**	424	1.00	474	1.06	371	1.01	383	**0.94**	407	0.96	338	**0.76**	388	1.05	426	**0.92**	409	0.96	449	1.03	3171	**0.95**
41–50	1717	1.04	1698	0.98	1906	1.08	1815	0.97	1584	0.98	1498	**0.92**	1443	**0.83**	1525	**0.81**	1507	**0.90**	1632	**0.91**	1578	**0.93**	1774	0.98	12,541	**0.91**
51–60	1592	1.06	1474	0.99	1581	1.07	1607	1.07	1339	0.98	1297	**0.89**	1311	0.97	1260	**0.87**	1418	0.97	1470	**0.90**	1384	**0.90**	1497	**0.91**	10,976	**0.92**
61–70	1841	1.01	1700	1.06	1694	1.04	1699	0.99	1345	**0.87**	1503	**0.88**	1564	**0.93**	1443	**0.86**	1517	**0.88**	1671	**0.90**	1501	**0.83**	1533	**0.85**	12,077	**0.87**
71-	2274	1.16	2000	1.14	2114	1.16	1992	1.04	1681	**0.86**	2059	**0.93**	2366	1.08	2063	**0.94**	2187	**0.94**	2405	**0.87**	2238	**0.87**	2277	1.01	17,276	**0.94**
Cormobidity*																										
None	5117	1.07	4655	0.99	5042	1.06	5056	1.02	4152	**0.94**	4218	**0.89**	4320	**0.92**	4153	**0.83**	4395	**0.92**	4806	**0.90**	4515	**0.91**	4830	**0.95**	35,389	**0.91**
1–2	2581	1.07	2416	1.08	2552	1.15	2369	1.01	2042	**0.90**	2348	**0.94**	2585	1.02	2326	**0.93**	2444	**0.94**	2612	**0.88**	2407	**0.85**	2538	**0.94**	19,302	**0.92**
3–4	183	1.29	159	1.38	121	**0.92**	153	1.13	119	**0.76**	169	1.18	178	1.09	144	1.05	170	1.05	179	1.01	175	**0.91**	156	1.08	1290	1.01
5 or more	3	–	8	–	4	–	9	–	7	–	5	–	8	–	6	–	8	–	7	–	13	–	6	–	60	1.22
Method of detection																										
Self detection	3688	1.07	3350	1.02	3695	1.10	3629	0.98	3286	**0.90**	3971	0.97	4339	1.07	3873	**0.95**	3905	**0.95**	4115	**0.89**	3639	**0.85**	3711	**0.92**	30,839	**0.94**
Screening (with symptom)	563	1.17	524	1.09	521	1.09	493	1.24	410	1.11	333	**0.87**	339	**0.78**	395	**0.89**	407	0.99	452	**0.93**	409	**0.91**	447	**0.94**	3192	**0.92**
Screening (without symptom)	2620	1.10	2405	1.03	2414	1.08	2459	1.08	1793	0.97	1463	**0.79**	1272	**0.71**	1363	**0.66**	1628	**0.80**	1941	**0.85**	1987	**0.88**	2273	**0.94**	13,720	**0.83**
Others	973	0.97	925	1.03	1044	1.06	962	0.97	800	**0.86**	933	**0.88**	1103	1.04	974	0.98	1055	1.11	1060	1.00	1046	1.04	1064	1.08	8035	1.00
Unknown	40	–	34	–	45	–	44	–	31	–	40	–	38	–	24	–	22	–	36	–	29	–	35	–	255	**0.72**
Clinical T status																										
Tis	1222	1.07	1077	1.02	1168	1.10	1151	1.08	901	**0.93**	920	**0.87**	943	**0.95**	862	**0.76**	946	0.97	1008	**0.88**	1052	1.01	1094	0.97	7726	**0.92**
T0	36	1.06	36	1.09	34	1.00	25	0.96	18	**0.49**	28	1.17	29	**0.88**	26	**0.74**	26	**0.79**	40	1.21	32	**0.82**	35	1.21	234	**0.89**
T1	3822	1.12	3433	1.00	3659	1.09	3610	1.04	2940	**0.94**	2988	**0.88**	3006	**0.87**	2934	**0.85**	3209	**0.90**	3480	**0.88**	3323	**0.87**	3602	0.96	25,482	**0.89**
T2	2123	1.00	2083	1.05	2194	1.06	2202	1.00	1933	**0.93**	2136	**0.94**	2377	1.07	2147	**0.92**	2171	**0.95**	2391	**0.90**	2126	**0.89**	2155	**0.91**	17,436	**0.94**
T3	256	1.24	196	1.03	219	1.18	218	**0.89**	182	**0.84**	222	1.01	259	1.11	213	1.00	226	**0.93**	225	**0.93**	183	**0.77**	219	0.96	1729	**0.94**
T4	317	0.96	313	1.11	355	1.14	294	**0.90**	275	**0.89**	366	1.03	382	1.09	341	0.96	347	0.99	365	1.03	304	**0.85**	329	0.97	2709	0.98
Unknown	108	–	100	–	90	–	87	–	71	–	80	–	95	–	106	–	92	–	95	–	90	–	96	–	725	**0.84**
Clinical N status																										
N0	6501	1.08	5961	1.03	6368	1.09	6225	1.02	5082	**0.92**	5393	**0.89**	5644	**0.94**	5302	**0.84**	5722	**0.93**	6247	**0.89**	5885	**0.89**	6246	0.96	45,521	**0.91**
N1	963	0.99	907	1.02	953	1.07	1006	1.05	909	0.98	945	0.96	1023	1.05	925	0.99	915	**0.90**	961	**0.88**	892	**0.88**	892	**0.88**	7462	**0.94**
N2	167	1.19	137	0.96	151	1.06	142	**0.86**	135	0.96	162	1.06	182	1.10	150	0.99	145	**0.95**	164	1.06	127	**0.77**	160	**0.88**	1225	0.97
N3	148	1.13	133	1.00	147	1.19	138	1.18	126	1.00	149	1.03	158	1.23	147	1.08	141	0.99	146	1.01	106	**0.86**	139	**0.95**	1112	1.02
Unknown	105	–	100	–	100	–	76	–	68	–	91	–	84	–	105	–	94	–	86	–	100	–	93	–	721	**0.84**
Clinical Stage																										
Stage 0	1215	1.07	1070	1.02	1163	1.10	1141	1.08	897	**0.93**	916	**0.88**	938	0.96	857	**0.76**	942	0.97	1004	**0.88**	1048	1.01	1086	0.97	7688	**0.92**
Stage I	3542	1.12	3197	1.01	3404	1.10	3355	1.03	2698	**0.94**	2741	**0.88**	2765	**0.87**	2694	**0.83**	2977	**0.90**	3245	**0.88**	3103	**0.87**	3371	0.97	23,594	**0.89**
Stage II	2333	1.02	2235	1.04	2352	1.07	2358	1.00	2090	**0.93**	2264	**0.92**	2532	1.06	2290	**0.94**	2305	**0.94**	2518	**0.89**	2253	**0.86**	2294	**0.92**	18,546	**0.93**
Stage III	545	1.08	487	1.07	536	1.11	523	0.97	449	**0.90**	581	1.04	611	1.10	534	**0.93**	546	0.96	565	0.97	471	**0.85**	516	**0.89**	4273	**0.95**
Stage IV	88	**0.92**	86	**0.91**	116	1.21	82	**0.90**	85	**0.89**	104	1.13	103	**0.93**	95	0.98	103	1.06	118	1.20	82	**0.77**	104	1.16	794	1.01
Unknown	161	–	163	–	148	–	128	–	101	–	134	–	142	–	159	–	144	–	154	–	153	–	159	–	1146	**0.84**
Breast cancer subtype																										
ER + and/or PgR +, HER2-	4940	1.12	4485	1.05	4773	1.11	4619	1.01	3789	**0.92**	4059	**0.90**	4240	**0.94**	4022	**0.85**	4340	**0.92**	4675	**0.89**	4455	**0.89**	4777	0.97	34,357	**0.91**
ER + and/or PgR +, HER2 +	755	1.02	677	1.03	743	1.15	738	1.03	663	1.04	679	1.01	696	0.98	687	0.98	650	1.02	721	1.00	608	**0.89**	643	**0.92**	5347	0.98
ER- and PgR-, HER2 +	431	1.03	393	1.03	383	0.96	436	1.13	397	1.06	357	**0.84**	440	1.14	353	**0.86**	396	**0.90**	411	**0.94**	375	**0.91**	352	**0.83**	3081	**0.93**
ER- and PgR-, HER2-	604	1.01	604	1.01	623	1.05	685	1.09	561	**0.93**	619	0.96	651	1.03	587	**0.89**	580	**0.87**	658	**0.81**	607	**0.92**	644	0.97	4907	**0.92**
Unknown	1154	–	1079	–	1197	–	1109	–	910	–	1026	–	1064	–	980	–	1051	–	1139	–	1065	–	1114	–	8349	**0.89**
Neoadjuvant endocrine therapy																										
Yes	354	1.17	292	1.09	325	1.16	314	1.02	291	0.97	356	1.14	356	1.03	299	**0.91**	307	1.07	337	1.03	277	**0.78**	241	**0.81**	2464	0.96
No	7512	1.07	6933	1.02	7375	1.08	7268	1.02	6019	**0.92**	6370	**0.90**	6724	**0.95**	6316	**0.87**	6697	**0.92**	7253	**0.89**	6821	**0.89**	7277	**0.95**	53,477	**0.91**
Unknown	18	–	13	–	19	–	5	–	10	–	14	–	11	–	14	–	13	–	14	–	12	–	12	–	100	**0.91**
Neoadjuvant chemotherapy																										
Yes	969	1.13	892	1.07	954	1.15	947	1.13	895	1.01	915	1.00	1032	1.18	928	1.04	889	**0.95**	952	**0.94**	733	**0.82**	851	**0.89**	7195	0.98
No	6897	1.07	6333	1.02	6746	1.08	6635	1.01	5415	**0.91**	5811	**0.89**	6048	**0.93**	5687	**0.85**	6115	**0.93**	6638	**0.89**	6365	**0.89**	6667	**0.95**	48,746	**0.91**
Unknown	18	–	13	–	19	–	5	–	10	–	14	–	11	–	14	–	13	–	14	–	12	–	12	–	100	**0.91**
Breast surgery																										
No	20	**0.83**	12	0.50	13	**0.52**	22	1.57	14	**0.78**	13	**0.72**	19	1.19	14	2.33	18	1.64	25	1.39	13	1.18	16	1.23	132	1.19
Breast conserving surgery	3581	1.09	3316	1.00	3503	1.08	3332	1.00	2703	**0.87**	2825	**0.89**	2899	**0.92**	2692	**0.80**	3034	**0.90**	3262	**0.83**	3075	**0.84**	3447	**0.95**	23,937	**0.87**
Mastectomy	4089	1.07	3728	1.05	4000	1.10	4048	1.03	3450	0.97	3718	**0.92**	3940	0.98	3716	**0.92**	3761	**0.95**	4085	**0.94**	3821	**0.92**	3888	**0.94**	30,379	0.94
Others	22	**0.69**	15	**0.48**	19	**0.66**	22	0.96	19	1.00	17	**0.68**	25	**0.83**	26	**0.90**	26	**0.90**	14	**0.61**	14	**0.82**	26	1.73	167	**0.89**
Unknown	172	–	167	–	184	–	163	–	135	–	167	–	208	–	181	–	178	–	218	–	187	–	153	–	1427	1.04
Axillary surgery																										
No	572	1.05	515	0.96	569	1.03	513	**0.95**	419	**0.75**	485	**0.84**	559	1.06	500	**0.95**	525	0.96	530	**0.84**	531	**0.88**	538	**0.91**	4087	**0.90**
SN	5256	1.10	4813	1.03	5102	1.10	5068	1.04	4201	0.96	4316	**0.90**	4417	**0.93**	4213	**0.84**	4558	**0.94**	4995	**0.89**	4674	**0.89**	5061	0.97	36,435	**0.91**
SN to Ax	596	0.99	578	1.05	587	1.04	557	**0.95**	461	**0.93**	511	**0.86**	581	0.99	533	**0.88**	531	**0.89**	570	**0.86**	615	1.00	598	0.99	4400	**0.93**
Ax	1164	1.02	1053	0.99	1170	1.10	1170	1.03	1021	**0.91**	1175	1.03	1237	1.03	1130	0.98	1126	**0.91**	1162	**0.91**	997	**0.81**	1088	**0.88**	8936	**0.93**
Others	110	1.08	101	1.16	98	1.01	101	**0.94**	73	**0.61**	75	**0.83**	76	**0.59**	62	**0.58**	84	**0.70**	110	**0.87**	96	**0.80**	80	**0.76**	656	**0.72**
Unknown	186	–	178	–	193	–	178	–	145	–	178	–	221	–	191	–	193	–	237	–	197	–	165	–	1527	0.99
Breast reconstruction																										
Yes	385	**0.68**	446	**0.81**	490	0.90	469	**0.79**	368	**0.79**	405	**0.70**	405	**0.82**	413	1.80	449	2.27	450	1.91	466	1.68	408	1.11	3364	1.19
No	7492	1.11	6786	1.05	7226	1.11	7112	1.04	5946	**0.93**	6329	**0.93**	6677	0.97	6210	**0.84**	6560	**0.89**	7146	**0.87**	6639	**0.86**	7112	**0.94**	52,619	**0.90**
Unknown	7	–	6	–	3	–	6	–	6	–	6	–	9	–	6	–	8	–	8	–	5	–	10	–	58	**0.83**
Adjuvant chemotherapy																										
Yes	1580	1.04	1476	1.00	1574	1.08	1640	1.01	1441	1.02	1492	0.99	1526	**0.94**	1432	**0.90**	1431	**0.91**	1584	**0.93**	1537	0.98	1553	0.97	11,996	**0.95**
No	5971	1.09	5481	1.04	5820	1.10	5649	1.02	4628	**0.90**	4963	**0.89**	5194	0.96	4864	**0.86**	5229	**0.92**	5609	**0.88**	5202	**0.86**	5608	**0.94**	41,297	**0.90**
Unknown	333	–	281	–	325	–	298	–	251	–	285	–	371	–	333	–	357	–	411	–	371	–	369	–	2748	**0.95**
Adjuvant radiotherapy																										
Yes	3344	1.08	3112	1.01	3256	1.09	3183	1.00	2564	**0.89**	2735	**0.94**	2642	**0.91**	2544	**0.82**	2725	**0.88**	2900	**0.85**	2654	**0.87**	2910	**0.94**	21,674	**0.89**
No	4158	1.07	3796	1.05	4090	1.10	4066	1.04	3469	**0.95**	3672	**0.89**	4035	0.99	3686	**0.90**	3866	**0.95**	4195	**0.92**	3954	**0.88**	4092	**0.95**	30,969	**0.93**
Unknown	382	–	330	–	373	–	338	–	287	–	333	–	414	–	399	–	426	–	509	–	502	–	528	–	3398	**0.95**

5% or more decrease is marked in bold

*ER* estrogen receptor, *PgR* progesterone receptor, *HER2* human epidermal growth factor receptor 2, *SN* sentinel node biopsy, *Ax* axillary node dissection

^*^Comorbidity includes cardiovascular diseases (such as myocardial infarction, chronic heart failure, peripheral vascular disease), cerebrovascular accident, chronic obstractive pulmonary disease, connective tissue disease, liver disease, diabetes mellitus, renal disease, hypertention, and other malignancies

### Changes in the patient demographics and detection methods of breast cancer before and during the COVID-19 pandemic

**Fig. 1:**
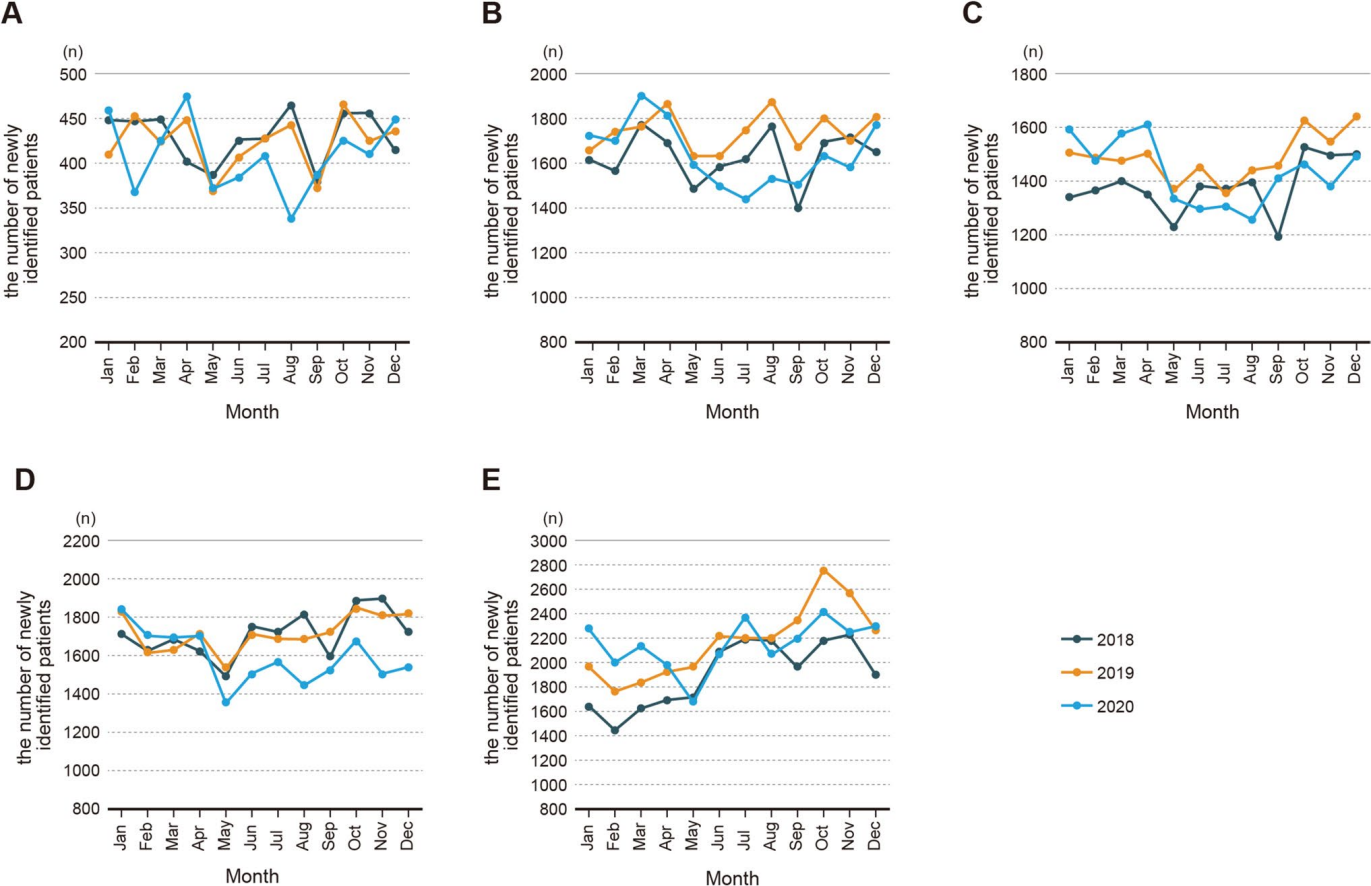
Monthly trends of the number of breast cancer cases in 2018, 2019, and 2020 by different age groups. **A** 20–40 years old, **B** 41–50 years old, **C** 51–60 years old, **D** 61–70 years old, **E** ≥ 71 years old

Table 2 and Table S1 shows the monthly trends of BC cases by patients and tumor characteristics. Examination of the annual trends within different age groups prior to the COVID-19 pandemic (2019) and comparing them with those during the year 2020 revealed that for the 20–40-year age group, a significant decline began in June 2020 (Table 2 and Fig. 1), reaching a peak decrease of 24% in August. Similarly, for the 41–50 and 51–60-year age groups, a significant reduction started in June and the most substantial drop occurred in August, reaching a peak decrease of 19% and 13%. Additionally, for the 61–70- and ≥ 71-year age groups, the decline began earlier than that in other age groups, starting in May, resulting in a 13% and 14% decrease. In the 61–70-year age group, no recovery was observed even by December, with patient numbers remaining 15% lower (Table 2 and Fig. 1). Examination of the rate of decrease over the 8-month period from May 2020 to December 2020, during the COVID-19 pandemic, showed that the 61–70-year age group exhibited the most substantial reduction, with a 13% decrease (Table 2).

The annual trends based on the number of comorbidities showed that in the group with ≤ 2 comorbidities, a decline started from May 2020 and persisted with no signs of recovery in December. However, in the group with ≥ 3 comorbidities, there was an initial decrease in May, but it quickly rebounded (Table 2 and Fig. S1).

**Fig. 2:**
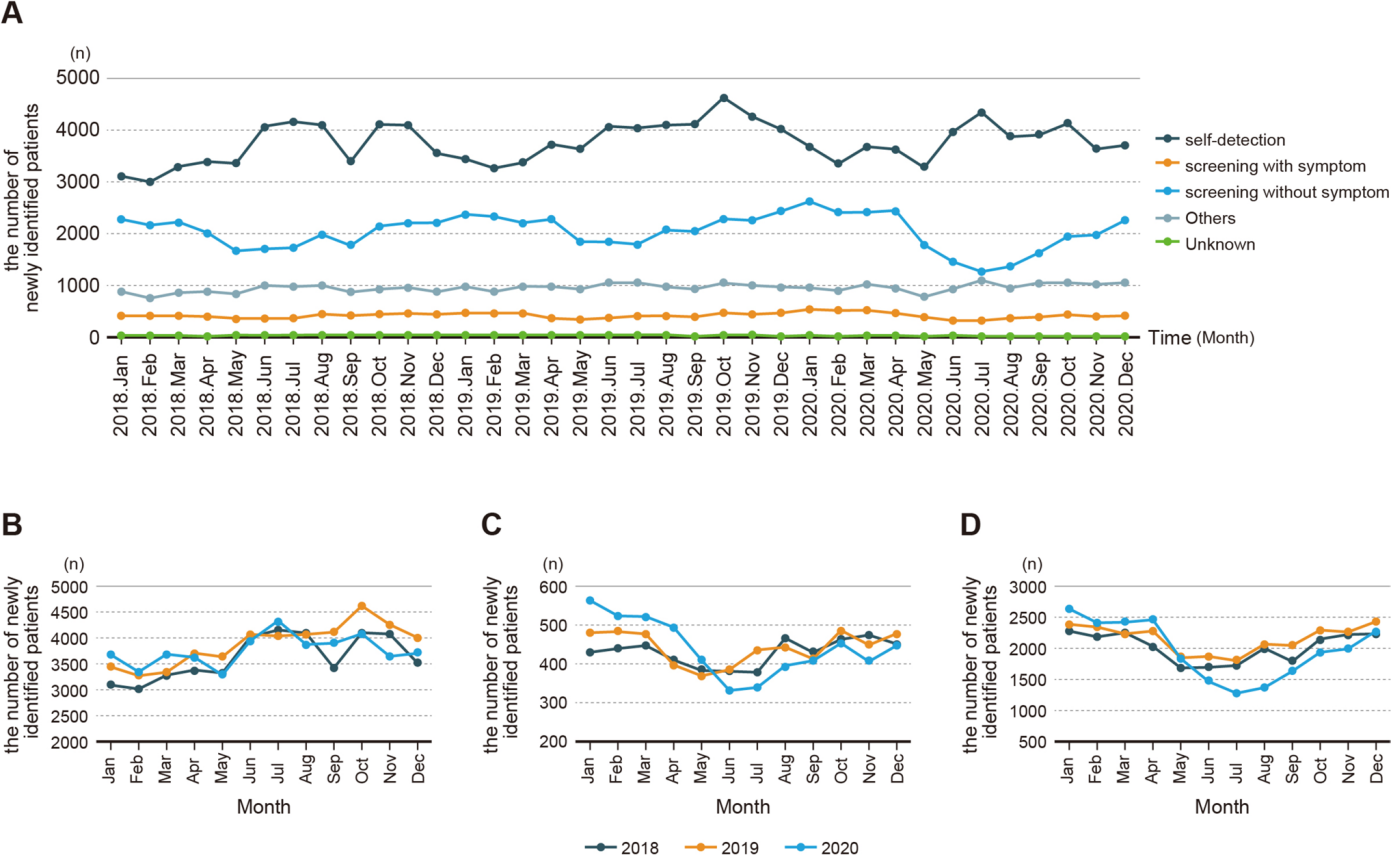
Changes of the number of breast cancer cases from January 2018 to December 2020 by different methods of detection (**A**). Monthly trends of self-detection (**B**), screening with symptom (**C**), screening without symptom (**D**)

Analysis of the annual trends for self-detected BC cases in 2020 showed a decline from May 2020, marking a 10% decrease. The most significant drop was noted in November, with a 15% reduction. For BC cases identified through screening that presented symptoms, a decline began in June, leading to a 13% decrease. The most significant decrease was seen in July, with a 22% reduction. The most significant change was observed in the detection of asymptomatic BC cases through screening. The decline began in June, marking a 21% decrease. This downward trend continued with a 29% decrease in July, a 34% decrease in August, and a 20% decrease in September. As of December, both self-detected BC and BC identified through screenings had not yet returned to the levels of the previous year (Table 2 and Fig. 2). Analysis of the decline rate over the 8-month period from May 2020 to December 2020 post-pandemic showed the most significant reduction in BC cases identified through asymptomatic screenings, with a 17% decrease. On the other hand, cases discovered through self-detection only saw a modest decrease of 6% (Table 2).

### Changes in clinical and pathological factors of breast cancer before and during the COVID-19 pandemic

**Fig. 3:**
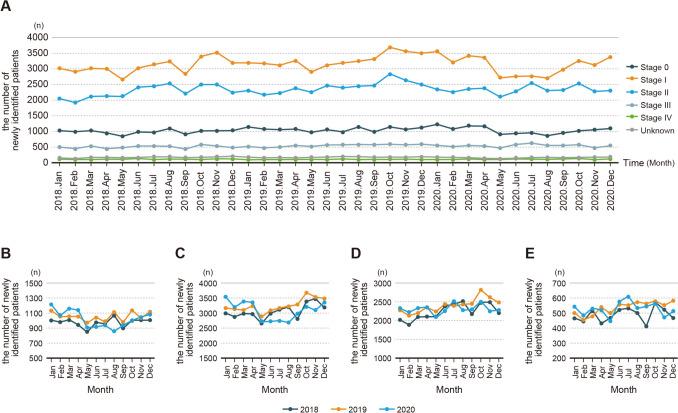
Changes of the number of breast cancer cases from January 2018 to December 2020 by clinical Stages (**A**). Monthly trends of Stage 0 (**B**), Stage I (**C**), Stage II (**D**), Stage III (**E**)

Regarding clinical stages, the decline for Stage 0 and Stage I began in May 2020, with a 7% and 6% decrease, respectively. Both stages reached their maximum in August, with Stage 0 decreasing by 24% and Stage I by 17% (Table 2 and Fig. 3). Following this, they gradually returned to levels similar to those of the previous year by December. Similarly, Stage II began to decline in May however showed no signs of recovery even by December. Throughout this period, most months consistently showed a decrease of approximately 10%. For Stage III, a decrease was observed in May with a 10% reduction (Table 2). Analysis of the rate of decrease over the 8-month period from May 2020 to December 2020, during the pandemic, showed a substantial reduction exhibited by cStage I, with a 11% decrease. The decrease for cStage II was 7%, and for cStage III was 5% (Table 2).

### Changes in breast cancer treatment before and during the COVID-19 pandemic

**Fig. 4:**
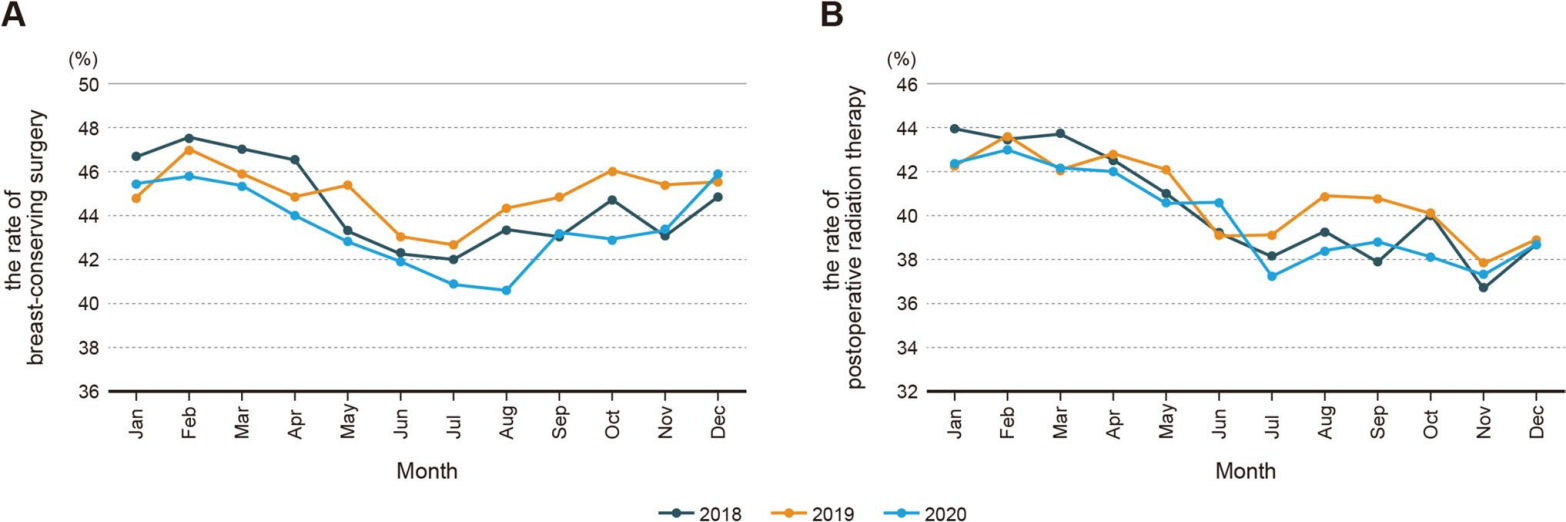
Monthly trends of the rate of breast cancer cases receiving breast-conserving surgery (**A**) and postoperative radiation therapy (**B**) in 2018, 2019, and 2020

In the context of breast surgery, the annual trend in breast-conserving surgery in 2020 showed a decline starting in May 2020, resulting in a 13% decrease. This decrease consistently continued throughout the period, with the peak decrease of 20% occurring in August. As of December, there was a trend toward recovery but the rates had not yet returned to the levels seen in the previous year. The proportion of mastectomy began to decline in June, with a peak reduction of 8% (Table 2 and Fig. 4). Examination of the reduction rate over the eight months period from May to December 2020 following the pandemic showed a noticeable 13% decrease compared with the previous year’s in cases involving breast-conserving surgery (Table 2). All axillary procedures began declining starting in May 2020, with their trends being affected by the status of the cN factors (Table 2). Although there was a breast reconstruction decrease of approximately 21–30% compared with that of the previous year since April 2020, it has been rapidly recovering and increasing compared with that of the previous year since September (Table 2). The yearly trend of postoperative radiation therapy began declining starting in May 2020, with a decrease of 11%. This decline remained consistent during the entire period (Table 2 and Fig. 4).

Analysis of the yearly trend of neoadjuvant chemotherapy by cStage showed that the proportion of neoadjuvant chemotherapy for Stage I was approximately 3% prior to the pandemic (Table S2 and Fig. S2A–E). However, in 2020, there was an unprecedented increase to 3.9% in May, 3.5% in June, 4.5% in July, and 4.4% in August, reaching new levels. From September, the rate returned to its prior level of approximately 3%, continuing the previous trend. Furthermore, no discernible impacts from the COVID-19 pandemic, either before or during the pandemic, were observed in Stage II and III (Table S2 and Fig. S2A–E). Examination of the yearly trend for neoadjuvant endocrine therapy by clinical Stage showed that the rate for Stage I was typically around 3–4% before the COVID-19 pandemic. However, in 2020, there was an unprecedented increase to 5.2% in May, 6.1% in June, 5.0% in July, and 5.2% in August, reaching new levels. From September, the proportion of neoadjuvant endocrine therapy was back to the previous level in the 3% range, following the prior trend. Furthermore, no discernible impacts from COVID-19 were observed either before or during the pandemic for Stage 0, II, and III (Table S2 and Fig. S2F–J).

### Changes in breast cancer subtypes before and during the COVID-19 pandemic

Regarding the subtypes of breast cancer, for the cases with ER + and/or PgR +, HER2 −, the number began to decrease in May 2020, showing an 8% reduction compared with that of the previous year. This downward trend continued almost consistently, reaching its peak with a 15% reduction in August. Signs of a recovery were finally observed in December. For cases with ER + and/or PgR +, HER2 +, there was no immediate change observed post-COVID-19 pandemic. The only significant decreases were noted in the months of November and December. For ER − and PgR −, HER2 + and ER − and PgR −, HER2 −, no consistent trend was observed during the study period. In the case of ER − and PgR −, HER2 +, there was a fluctuation from a 17% decrease to a 14% increase compared with that of the previous year of the pandemic. For ER − and PgR −, HER2 −, there was a fluctuation from a 19% decrease to a 3% increase (Table 2 and Fig. S3). Examination of the rate of decline over the 8-month period from May 2020 to December 2020, during the pandemic, showed the most reduction in ER + and/or PgR +, HER2 −, with a 9% decrease compared with that of the previous year. In contrast, ER + and/or PgR +, HER2 + showed a smaller decrease of 2% (Table 2).

## Discussion

Using real-world data from roughly 300,000 individuals across the country, our study has shown how the diagnosis and treatment of BC change during emergencies like the COVID-19 pandemic. This investigation using big data will be crucial for future crisis preparation, offering preliminary information for evaluating the consequences of suspending BC screenings and deciding on necessary BC treatments during medical scarcity.

The trend in the number of patients with BC in the years leading up to 2019 demonstrated an annual growth rate of approximately 2–3% [7, 13, 14]. Examination of the monthly changes in the number of patients with BC post-pandemic showed a 4–13% decrease compared with that of the previous year. Specifically, during the eight months period from May 2020 to December 2020, following the onset of the COVID-19 pandemic, there was a 9% decrease compared with the same period in the previous year. When considering the natural rate of increase, these findings suggest a concern of delayed diagnosis in approximately 10% of patients with BC. A few reports have been published, that modeled and assessed the impact of delayed diagnosis on patient outcomes [6, 15]. A delay of 6 months in BC treatment is reported to lead to a 0.5% increase in the mortality rate from BC. However, this increase is considered relatively minor, as it is < 1% [15]. The impact on prognosis in these studies is based solely on simulation, and therefore, long-term follow-up will be essential to accurately assess future outcomes. In the present study, it was observed that a decrease in the rate of screening detected BC have led to a reduced numbers of patients in the early stages, specifically Stage 0 and Stage I. However, in contrast, there was no significant decrease in patients with advanced Stage BC. The prognosis for Stage 0 and Stage I BC is reported to be exceptionally good, suggesting that any delay in cancer detection or BC treatment within this group may have a relatively limited impact on prognosis. Moreover, it is hypothesized that only a small percentage of patients with Stage II or higher BC have experienced a delay of ≥ 6 months in initiating treatment. However, to truly assess these impacts, future prognosis analyses must be conducted over an adequately extended observation period.

Investigating which patient demographics are impacted by the pandemic is crucial for devising strategies for future pandemic scenarios. This is particularly relevant in the context of COVID-19. Examination of the data by age group showed that the initial noticeable impact immediately following the declaration of a state of emergency in May 2020 was observed in the older patient demographics, specifically those aged 61–70 and those aged ≥ 71 years. While the numbers for other age groups remained relatively the same as the previous year, there was a 13% decrease in the age group of 61–70 and a 14% decrease in the age group of ≥ 71. This can be attributed to data indicating a higher rate of severe illness due to COVID-19 among the elderly, leading to the inference that older individuals were more hesitant to seek medical care. Conversely, in the age group of 20–40 years, there was a notable decrease of 24% from the previous year during the peak of what is referred to as the second wave in August 2020. However, no significant changes were observed during other periods throughout the study compared with that of the previous year. The impact was still evident as of December 2020, especially in the 51–60- and 61–70-years age groups. These age groups experienced a significant decrease throughout the study period. The most substantial reduction during the eight months period following the pandemic was observed in the 61–70-year age group. This suggests that the effect of avoiding medical screenings was most pronounced in this age group. In terms of comorbidities, there was a notable decrease in the number of patients with 3–4 comorbidities in May 2020, immediately after declaring of a state of emergency, after which the numbers swiftly rebounded. This can be attributed to similar reasons as those previously mentioned for the senior population.

When examining the data based on the detection method, it is naturally anticipated that a decline in screening attendance would lead to these results. However, the most substantial reduction was noted in patients identified through screenings where they did not report any symptoms themselves. From May to December 2020, there was a 17% decrease in the number of such patients compared with the same period in the previous year. Specifically, there were 13,720 cases in 2020, down from 16,583 cases in the previous year. This decrease was more pronounced than the 6% decrease in self-detection (from 32,881 cases to 30,839 cases), and the 8% decrease in cases identified through screenings with self-reported symptoms (from 3453 cases to 3192 cases). The effects of decreased screening participation were as predicted. However, the initial concern about a significant drop in medical facility visits by patients with self-reported symptoms may not have been as substantial as initially thought, given the 6% decrease in self-discovered BC cases. These effects were also apparent in the cT, cN, and cStage classifications, with significant reductions in cases classified as cT1, cN0, cStage 0, and cStage I. This corresponds with the observed decrease in BC cases usually detected through screenings. The timing is unclear regarding when these groups of BC cases that would have otherwise been diagnosed will be diagnosed and treated in the future, and it is necessary to closely monitor subsequent changes in the number of cT, cN, and cStage-classification BC cases.

It is crucial to investigate whether the current pandemic has negatively impacted patients with BC. In other words, if the COVID-19 pandemic has unnecessarily imposed changes in BC treatment, then improvements should be considered. In breast surgery procedures, there was a notable decrease in breast-conserving surgery. This could be due to a decrease in early-stage BC cases. It is believed that another major reason for the decrease in early-stage BC is the significant reduction in cases that did not involve radiation therapy compared with that of the previous year. However, it is also possible that this reduction could be attributed to patients choosing mastectomy to avoid radiation therapy. In this context, it is essential for both healthcare professionals and patients to understand that breast-conserving strategy (breast-conserving surgery followed by radiation therapy) can be safely implemented with appropriate infection control measures. In terms of drug treatment, a more pronounced decrease was observed in cases without neoadjuvant endocrine therapy or (neo) adjuvant chemotherapy. This suggests that the probability of patients abstaining from drug treatment is extremely low. Furthermore, the reduction in early-stage BC cases is also considered to be a contributing factor to these results. A notable change in BC treatment due to the COVID-19 pandemic, as observed in this study, was the rise in the use of neoadjuvant endocrine therapy or chemotherapy for Stage I BC patients despite no changes being made to the clinical guidelines. This increase was particularly evident in the immediate months following the onset of the pandemic, from May to September. This period aligns with a time when healthcare systems were not yet fully established, leading to a significant amount of trial and error in the treatment and care of patients with BC. This data shows that during this period, strategies at the clinical level were implemented to avoid treatment delays, using neoadjuvant endocrine therapy or chemotherapy. In this context, it is believed that healthcare institutions made accurate decisions to minimize potential harm to BC patients.

This study revealed a temporary delay in BC diagnosis and changes in treatment; however, evaluating the long-term impact on patient outcomes including overall survival will require further investigation with an adequate follow-up. Another important point is that when analyzing the impact of COVID-19, particularly in elderly populations and those with underlying health conditions, it is essential to consider socioeconomic and geographical factors. The inability to include such variables represents one of the limitations of this study. If possible, we want to provide specific recommendations or frameworks in a future pandemic, however as we do not currently have prognostic data, it remains unclear how we should act in a pandemic in terms of not affecting cancer diagnosis and treatment.

In conclusion, from this study using data from all of Japan, we have identified the patient groups that are more vulnerable to the effects of a pandemic. Specifically, these groups are at a higher risk of seeing a decline in BC diagnosis and treatment. Furthermore, the changes in BC care that occur during emergencies have also been revealed. There exists a subset of cases where diagnosis is delayed to a certain degree. It is vital to ascertain when these cases will be diagnosed, and whether the delay affected the prognosis or results in any disadvantages in the treatment of BC. It is important to wait for the results and evaluate the comprehensive effect that the COVID-19 pandemic has had on the diagnosis and treatment of BC. 

## Supplementary Information

Below is the link to the electronic supplementary material.

Supplementary file1 (DOCX 778 KB)

Supplementary file2 (XLSX 30 KB)

## Data Availability

Unfortunately, these raw data are not available freely.
