# Supplementary material for: Impact of the COVID-19 pandemic on breast cancer diagnosis and treatment trends in Japan
Source: Breast Cancer. 2025 May 12;32(5):947–59. doi: 10.1007/s12282-025-01718-2 (PMC12394362; doi:10.1007/s12282-025-01718-2)
Supplement: Supplementary file 1 — Supplementary file1 (DOCX 778 KB) [file 12282_2025_1718_MOESM1_ESM.docx]

**
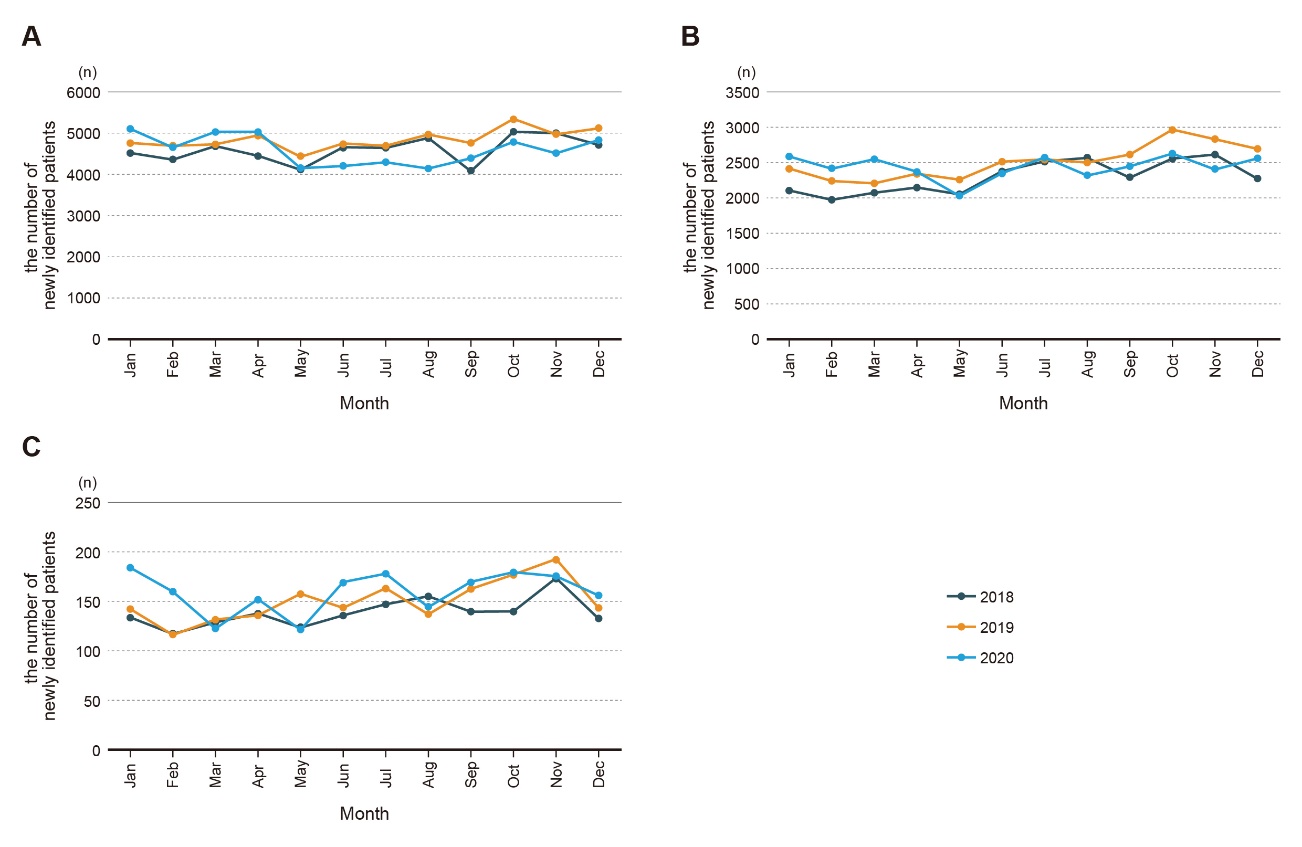
Supplementary Figure 1**

**Supplementary Figure 1**. Monthly trends of the number of breast cancer cases in 2018, 2019, and 2020 by different numbers of comorbidity. **A**) none, **B**) 1-2, **C**) 3-4.

**Supplementary Figure 2**.


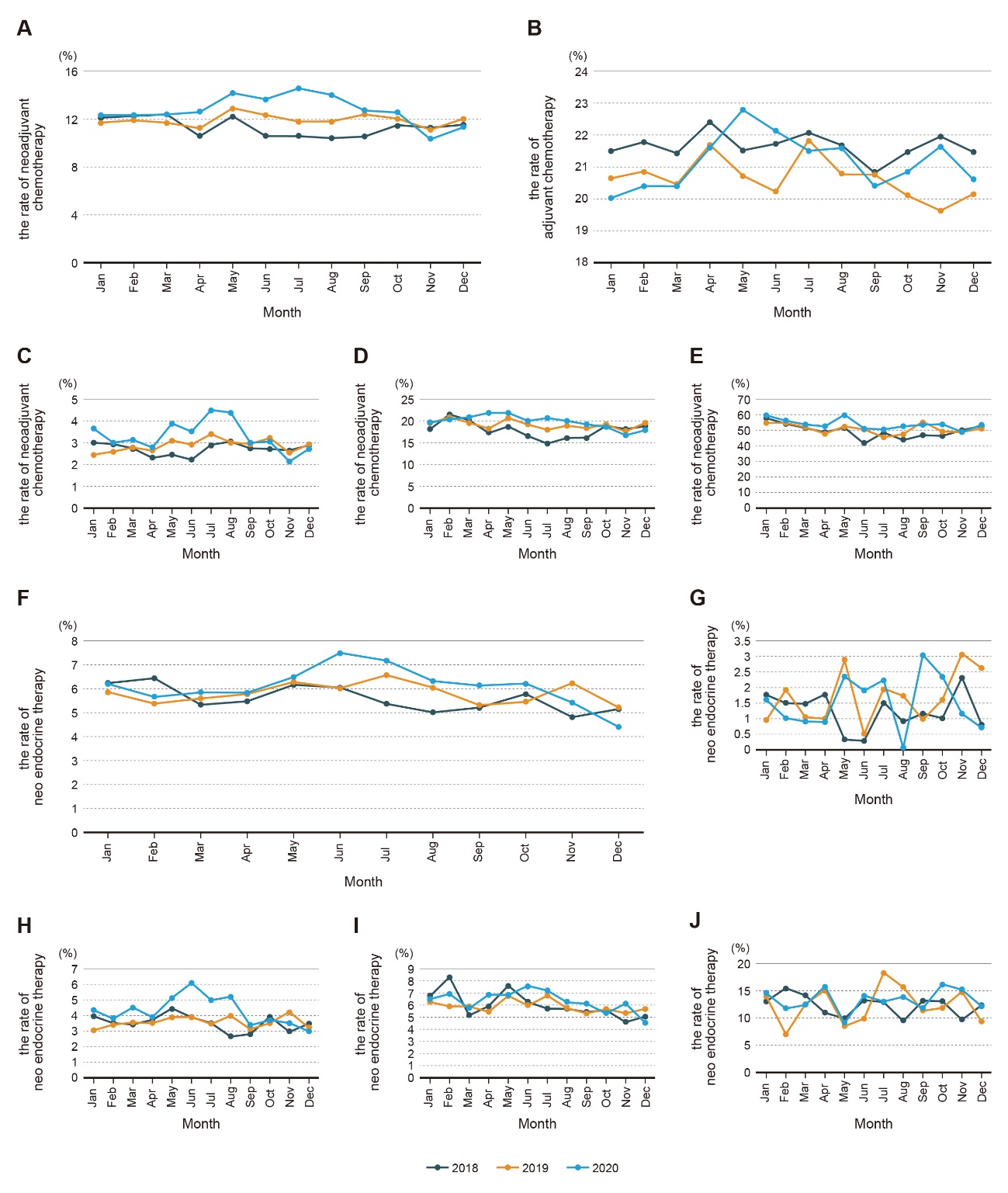
**Supplementary Figure 2**. Monthly trends of the rate of breast cancer cases receiving neoadjuvant chemotherapy (**A**) and adjuvant chemotherapy (**B**) in 2018, 2019, and 2020. **C**, **D**, and **E** indicate the rate of clinical Stage I, II, and III breast cancer cases receiving neoadjuvant chemotherapy. And monthly trends of the rate of breast cancer cases receiving neoadjuvant endocrine therapy (**F**) in 2018, 2019, and 2020. **G**, **H**, **I**, and **J** indicate the rate of clinical Stage 0, I, II, and III breast cancer cases receiving neoadjuvant endocrine therapy.

**Supplementary Figure 3**.


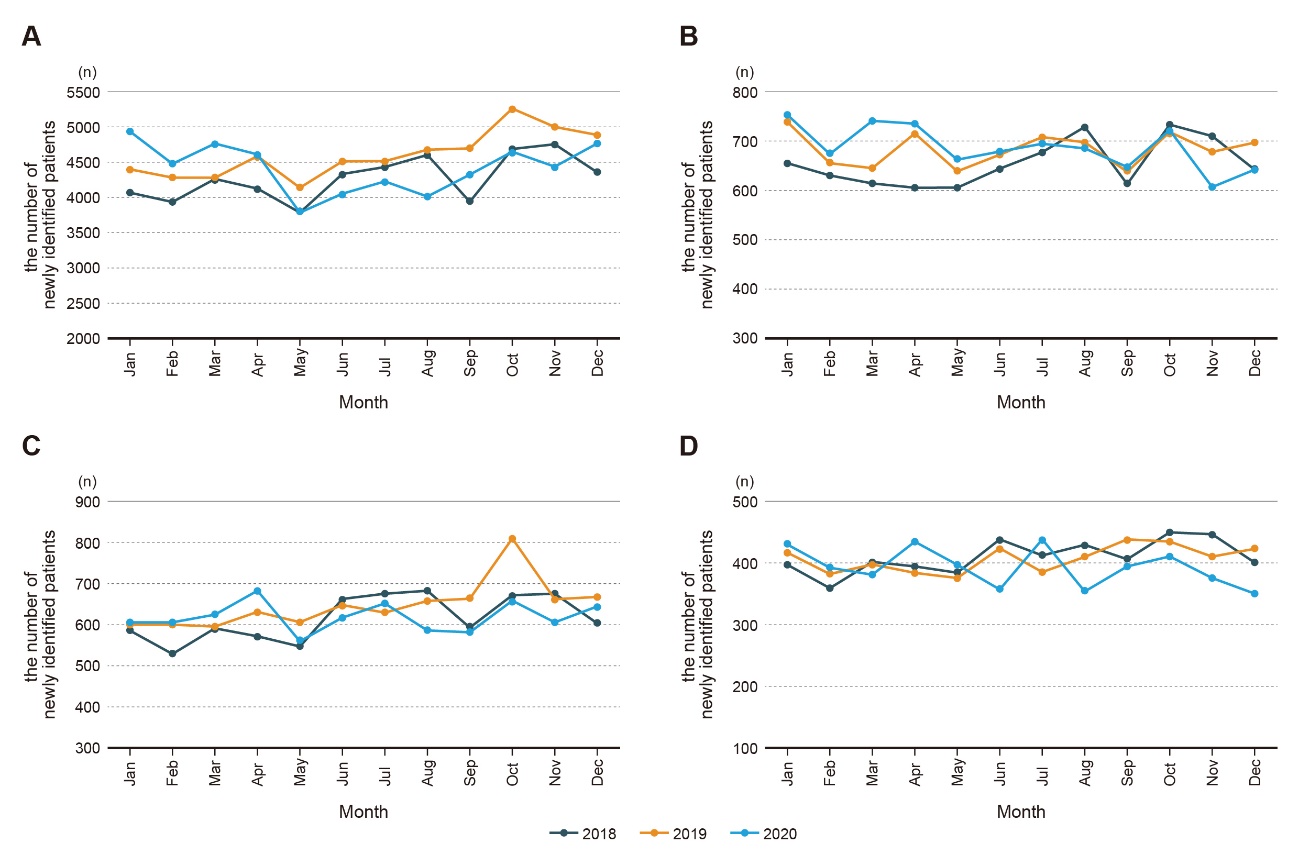


**Supplementary Figure 3**. Monthly trends of the number of breast cancer cases in 2018, 2019, and 2020 by different breast cancer subtypes. **A**) ER+ and/or PgR+, HER2−, **B**) ER+ and/or PgR+, HER2+, **C**) ER− and PgR−, HER2+, **D**) ER− and PgR−, HER2−.
